# *Phanerochaete sordida* as a cause of pulmonary nodule in an immunocompromised patient: a case report

**DOI:** 10.1186/s12879-017-2244-9

**Published:** 2017-02-10

**Authors:** Naoki Watanabe, Kiyofumi Ohkusu, Masaya Okuda, Osamu Imataki, Tomoya Ishii, Kiyoshi Negayama, Akira Tadokoro, Nobuyuki Kita, Takehiro Takagi, Nobuhiro Kanaji, Norimitsu Kadowaki, Shuji Bandoh

**Affiliations:** 1 Department of Internal Medicine, Hematology, Rheumatology and Respiratory Medicine, Faculty of Medicine, Kagawa University, 1750-1 Ikenobe, Miki-cho, Kita-gun, Kagawa, 761-0793 Japan; 2 Department of Microbiology, Tokyo Medical University, 6-1-1 Shinjuku, Shinjuku-ku, Tokyo, 160-8402 Japan; 3 Department of General Thoracic, Breast and Endocrinological Surgery, Faculty of Medicine, Kagawa University, 1750-1 Ikenobe, Miki-cho, Kita-gun, Kagawa, 761-0793 Japan; 4 Department of Clinical Laboratory, Faculty of Medicine, Kagawa University, 1750-1 Ikenobe, Miki-cho, Kita-gun, Kagawa, 761-0793 Japan

**Keywords:** *Phanerochaete sordida*, Pulmonary nodule, Broad-range polymerase chain reaction, Fungal infection

## Abstract

**Background:**

*Phanerochaete sordida* is a species of wood rotting fungus, which can degrade lignin, cellulose and hemicellulose contained in wood and other hard-to-biodegrade organic substances. However, to date, there have been no other reports demonstrating that *P. sordida* can infect humans.

**Case presentation:**

A 66-year-old Japanese man presented for a mass increasing in size on his left thigh. He had been suffering from rheumatoid arthritis for 18 years and chronic obstructive pulmonary disease for 20 years, for which he was being treated with 5 mg/day prednisolone and 8 mg/week methotrexate. The mass resection was performed two months later, and was diagnosed as malignant fibrous histiocytosis. However, a computed tomography examination for tumor recurrence after surgery showed a newly emergent pulmonary nodule. We therefore decided to resect the nodule by thoracoscopic procedure. Histopathological examination of the excised specimen showed that the lesion was a granuloma, with necrotic tissue and clumping of *Aspergillus*-like hyphae. Therefore, the nodule was diagnosed as a fungal infection and tissue specimens were cultured microbiologically. However, fungal growth was not observed. We consequently performed genetic analysis using a broad-range polymerase chain reaction. The 28S rRNA sequence demonstrated 100% homology with *P. sordida* using the NCBI BLAST program against the GenBank DNA databases.

**Conclusions:**

Using broad-range polymerase chain reaction, we identified *P. sordida* as the causative agent of a pulmonary nodule. These findings indicate that *P. sordida* may be an additional opportunistic causative organism of pulmonary infection in immunocompromised patients.

## Background

Over 300 species of fungi are regarded as pathogenic to humans, with the number of fungal species reported to cause opportunistic infections still increasing [1]. Notably, no specific gene or phenotype has been identified as a marker of pathogenicity in humans. *Phanerochaete sordida* is a species of wood-rotting fungus distributed worldwide [2]. This fungus belongs to the family of white-rot fungi that have the ability to degrade the lignin, cellulose and hemicellulose contained in wood. White-rot fungi are often edible, including shiitake mushrooms (*Lentinus edodes*), oyster mushrooms (*Pleurotus ostreatus*) and king trumpet mushrooms (*Pleurotus eryngii*). Enzymes found in white-rot fungi have been reported to break down hard-to-degrade compounds such as dioxins. Fungal enzymes are therefore also being assessed for use in bioremediation. Although several *P. sordida* related studies have been conducted, *P. sordida* is generally regarded as unable to induce diseases in humans and animals.

This report describes a patient with a pulmonary nodule induced by *P. sordida*. To our knowledge, this is the first report showing that *P. sordida* is pathogenic in humans.

## Case presentation

A 66-year-old Japanese man became aware of a mass on his left posterior surface in March 2014. Within a month, the mass had increased in size, and he began to experience left femoral pain. His symptoms gradually worsened, and the mass was excised partially in May 2014. Histopathological investigation at that time resulted in a diagnosis of malignant fibrous histiocytoma. As computed tomography (CT) scans of the patient’s entire body showed multiple pulmonary nodules, he was referred to our hospital for further examination and treatment.

The patient’s previous medical history included rheumatoid arthritis for 18 years and chronic obstructive pulmonary disease for 20 years, for which he was being treated with 5 mg/day prednisolone and 8 mg/week methotrexate. He was an ex-smoker, with a 60 pack-year history, and had been treated for 5 years with 1.0 L/min home oxygen therapy. He had a history of treatment with the non-steroidal anti-inflammatory drug (NSAID) loxoprofen.

**Fig. 1:**
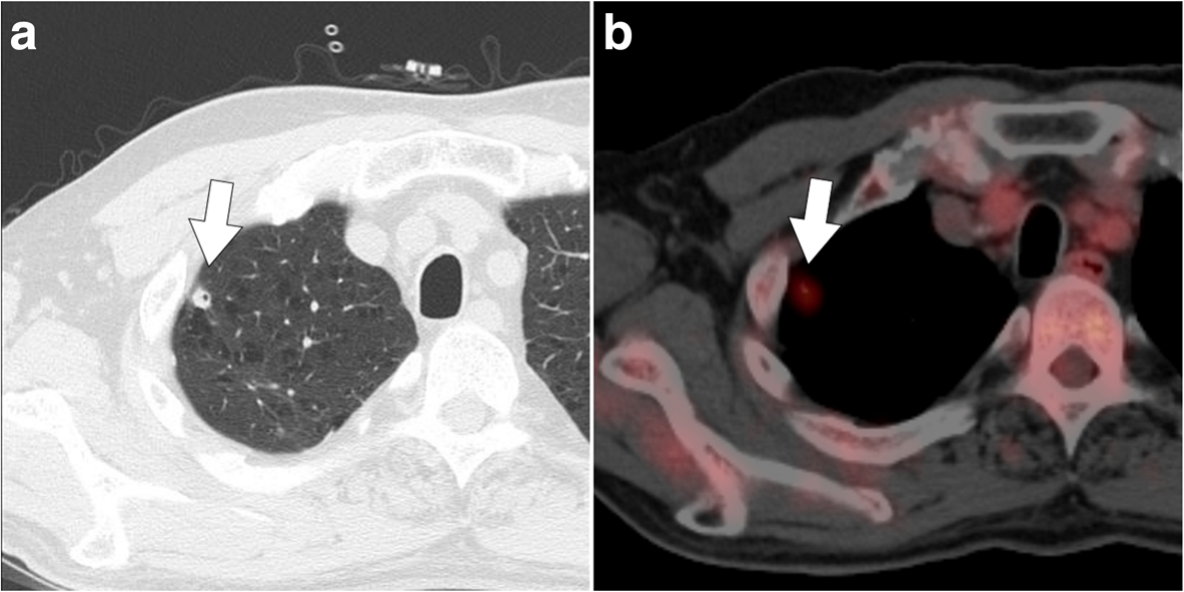
Computed tomography (CT) and fluorodeoxyglucose positron emission tomography (FDG-PET) findings. **a** Chest CT, showing a pulmonary nodule (*arrow*) in the right apical portion of the lung. **b** FDG-PET, showing accumulation of fluorodeoxyglucose in the pulmonary nodule (*arrow*)

On admission, the patient’s height was 164.5 cm and his weight was 57.4 kg. His blood pressure was 128/96 mmHg; his heart rate was 76 beats/min and regular; his oxygen saturation was 97% while receiving 1.0 L/min oxygen; and his body temperature was 35.6 °C. Physical examination showed no remarkable findings, except for the left femoral mass. Chest CT revealed an additional nodule in the right apical portion of the lung, with no change in size of the preexisting nodules (Fig. 1-a). All the nodules were under 3 to 5 mm in size. Positron emission tomography showed accumulation of fluorodeoxyglucose (SUVmax: 2.39) only in the nodule newly identified on CT (Fig. 1-b).

The left femoral tumor was excised completely in July 2014. The patient’s postoperative course was uneventful. Because postoperative chemotherapy was indicated only if the newly developed nodule was a metastatic tumor, he underwent diagnostic wedge resection of the right upper lobe under video-assisted thoracoscopy in August 2014, with tissue samples obtained aseptically. However, this procedure was not able to excise all lung nodules.

**Fig. 2:**
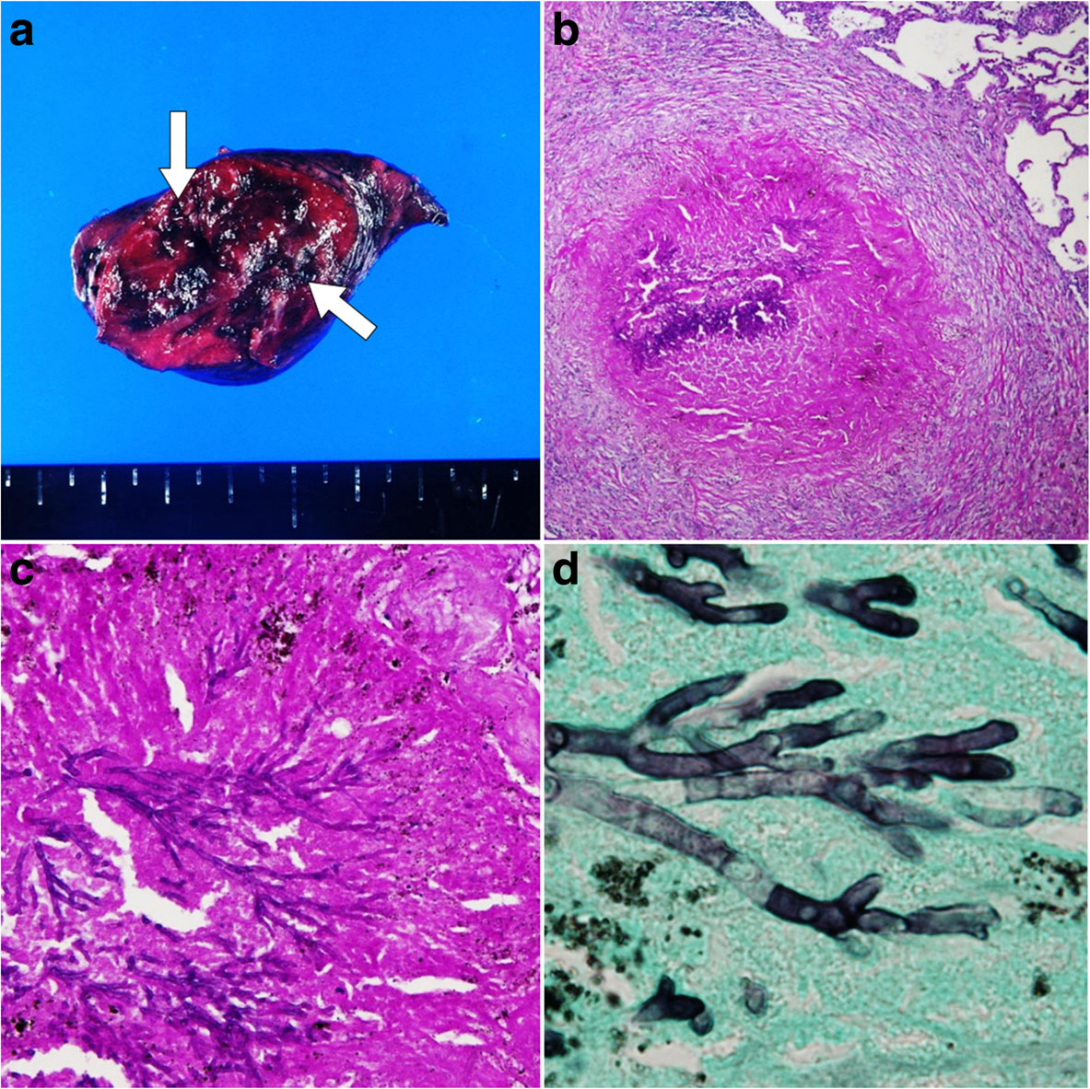
Histopathological findings of the pulmonary nodule in this patient. **a** Macroscopic examination of the nodule, showing a *black node* with a *yellowish white* interior (*arrow*). **b** Periodic acid-Schiff (PAS) staining, showing a granuloma with clumping of hyphae. Fibrotic changes and invasion of inflammatory cells are seen at the periphery of the granuloma (original magnification × 20). **c** PAS staining, showing a granuloma with necrotic tissue and clumping of hyphae (original magnification × 40). **d** Grocott staining, showing septate hyphae (original magnification × 100)

Macroscopic findings revealed a black node with a yellowish white interior (Fig. 2-a). Histopathological examination of the specimen showed that the lesion was a granuloma, with necrotic tissue and clumping of *Aspergillus*-like hyphae. Grocott’s staining and periodic acid-Schiff (PAS) staining (Fig. 2-b, c, d) were performed. The periphery of the granuloma showed fibrotic changes and invasion of inflammatory cells, including lymphocytes, histiocytes and multinucleated giant cells. In addition, the thin granuloma layer was surrounded by necrotic tissue. There was no evidence of malignancy. These findings indicated a granuloma caused by fungal infection.

Tissue specimens were cultured microbiologically on potato dextrose agar medium at 37 °C. However, fungal growth was not observed, even after 4 weeks. Gene analysis using broad-range polymerase chain reaction (PCR) was performed to identify the pathogen. The 28S rRNA gene of stored tissue samples was amplified and sequenced. Analysis of the 28S rRNA gene of this specimen using the NCBI BLAST program comparing against the NCBI GenBank DNA database revealed 100% homology to *P. sordida*. The patient was diagnosed with pulmonary mycosis caused by *P. sordida*.

Because the nodule was not a metastasis of the malignant tumor, the patient underwent localized postoperative radiation therapy of his femoral region to prevent local recurrence. As no antifungal drugs have been tested against *P. sordida*, the patient is being carefully monitored every 2 months. Currently, 6 months after the lung surgery, there has been no evidence of exacerbation of lung nodules.

## Discussion

*P. sordida* is a white-rot wood fungus, the only type of microbe that can effectively degrade lignin, a key component of wood. As they contain enzymes that degrade lignin, white-rot fungi are utilized for bioremediation. The YK-624 strain of *P. sordida* is one of a series of strains collected on Yakushima Island, Japan, that is both highly active and selectively degrades lignin [3]. To date, *P. sordida* has not been reported to be pathogenic in humans or animals. The US Centers for Disease Control and Prevention has reported that *P. sordida* has a biosafety level of 1, indicating that this microbe does not induce diseases in humans or animals [4].

Nevertheless, we found that this patient was infected by *P. sordida*, probably because he was in an immunocompromised state. He had a long history of rheumatoid arthritis and was being treated with prednisolone and methotrexate. Moreover, he had been diagnosed with malignant fibrous histiocytoma. Furthermore, he had a 20-year history of chronic obstructive pulmonary disease, which may have caused airflow stasis in his lower respiratory airways. Being an ex-smoker and on home oxygen therapy may also have contributed to infection with this normally non-pathogenic fungus. *P. sordida* has been shown to metabolize the NSAIDs, diclofenac and mefenamic acid [5], a biochemical property that can contribute to the establishment of local *P. sordida* infections in humans. That is, the metabolism of NSAIDs at local infection sites can enhance the growth of *P. sordida* by suppressing the local inflammatory reaction of host immune cells.

*P. sordida* is unable to grow at the normal human body temperature of 37 °C. If this fungus is aspirated and migrates to the bronchial membrane in patients with chronic obstructive pulmonary disease, *P. sordida* may be able to grow slowly but not over a certain size. *P. sordida* infection in humans may therefore be characterized by small-sized nodules on CT images and slight granuloma reactions in situ.

Histopathological examination and genetic analysis of the pulmonary specimen resected from our patient resulted in a diagnosis of pulmonary mycosis induced by *P. sordida*. The inability to cultivate the fungus from the tissue specimen was likely due to the low viability of the fungus or the presence in the tissue specimen of dead or damaged fungi. In addition, the American Type Culture Collection (ATCC) recommends that all the *P. sordida* strains it provides be cultivated in potato dextrose agar medium at 24 °C for approximately 4 weeks [6]. Differences in temperature may result in unsuccessful cultivation [7]. Notably, the temperature at which the tissue specimen from this patient was cultured at therefore differed from the ideal conditions. Thus, the absence of other reports on the pathogenicity of *P. sordida* may also be a result of cultivation under suboptimal conditions. Accordingly, genetic analysis may enable identification when DNA can be collected, and is very useful for identification of pathogens when cultivation is unsuccessful.

To determine the antifungal susceptibility of this strain, we obtained the reference strain *P. sordida* YK-624 (ATCC 90872) and conducted an antifungal susceptibility test using the microdilution method according to the guidelines of the Clinical and Laboratory Standards Institute (M38A). Although the growth of this strain was observed in potato dextrose agar medium, it did not form spores. Therefore, we could not complete the susceptibility test. Subsequently, we conducted antifungal susceptibility testing using the agar plate dilution method with voriconazole and liposomal amphotericin B. Voriconazole was active against this strain at concentrations ≥2.5 μg/mL. In contrast, 10 μg/mL liposomal amphotericin B did not inhibit the growth of this strain. Therefore, it may be necessary to establish a method for antifungal susceptibility testing of filamentous fungi not dependent on sporulation. Notably, at least for *P. sordida* YK-624, voriconazole was found to have antimicrobial activity. To our knowledge, there have been no previous reports describing *P. sordida* infections in humans. Importantly, if fungi cannot be cultivated, despite the presence of hyphae on histopathological examination and smears, they may be successfully cultivated under different conditions, or identified by genetic analysis.

Although the clinical significance of *P. sordida* infection has not been clarified, this fungus may be pathogenic in humans. Assessment of additional infected patients is required to determine the characteristics of infection by this rare opportunistic pathogen. Currently, we are still monitoring the patient’s lung nodules for an increase in size or number. Fortunately, an increase in lung nodules has not been seen thus far. However, recurrence of opportunistic infections may occur frequently in immunosuppressed patients. Until the antifungal susceptibility is known, it is worth considering the use voriconazole as an antifungal agent based on the results of our susceptibility testing on a *P. sordida* reference strain.

## Conclusions

Using broad-range PCR, we identified *P. sordida* as the causative agent of a pulmonary nodule in a patient. Our case report indicates that *P. sordida* may be an additional opportunistic causative organism of pulmonary infection in immunocompromised patients. Importantly, molecular methods may be helpful in identifying pulmonary *P. sordida* infections in this patient population.

## Abbreviations

CT: Computed tomography
NSAIDs: Non-steroidal anti-inflammatory drugs
PAS: Periodic acid-Schiff
PCR: Polymerase chain reaction
